# Prognostic Role of Serum Antibody Immunity to p53 Oncogenic Protein in Ovarian Cancer: A Systematic Review and a Meta-Analysis

**DOI:** 10.1371/journal.pone.0140351

**Published:** 2015-10-09

**Authors:** Marica Garziera, Marcella Montico, Ettore Bidoli, Simona Scalone, Roberto Sorio, Giorgio Giorda, Emilio Lucia, Giuseppe Toffoli

**Affiliations:** 1 Experimental and Clinical Pharmacology Unit, Centro di Riferimento Oncologico (CRO), IRCCS, Aviano National Cancer Institute, via F. Gallini 2, 33081, Aviano, (PN), Italy; 2 Epidemiology and Biostatistics Unit, Centro di Riferimento Oncologico (CRO), IRCCS, Aviano National Cancer Institute, via F. Gallini 2, 33081, Aviano, (PN), Italy; 3 Department of Medical Oncology, Centro di Riferimento Oncologico (CRO), IRCCS, Aviano National Cancer Institute, via F. Gallini 2, 33081, Aviano, (PN), Italy; 4 Department of Gynecological Oncology, Centro di Riferimento Oncologico (CRO), IRCCS, National Cancer Institute, via F. Gallini 2, 33081, Aviano, (PN), Italy; Sapporo Medical University, JAPAN

## Abstract

**Objective:**

Serum p53 autoantibodies (p53-AAbs) are the product of an endogenous immune response against p53 overexpression driven by the ovarian tumour. The p53-AAbs are detectable only in a subset of patients. To date, the evidence of an association between the presence of p53-AAbs and ovarian cancer outcomes has been poorly investigated.

**Methods:**

A systematic literature search was performed to identify eligible studies investigating the association of serum p53-AAbs and overall survival (OS) and disease free survival (DFS). Associations between presence of serum p53-AAbs and baseline tumour characteristics were also evaluated. Pooled hazard ratios (HRs) and corresponding 95% confidence intervals (CI) were computed to estimate the prognostic impact of serum p53-AAbs. Heterogeneity between studies was assessed.

**Results:**

A total of 583 patients (7 studies) for OS and 356 patients (4 studies) for DFS were included in the meta-analysis. Presence of p53-AAbs was not associated to OS (pooled uni- multivariate HR = 1.09; 95% CI: 0.55–2.16), and a large heterogeneity was found. When only multivariate HRs were pooled together (4 studies), presence of p53-AAbs was significantly associated to a better OS (pooled HR = 0.57; 95% CI: 0.40–0.81), and no significant heterogeneity was observed. A reduced DFS was associated to p53-AAbs (pooled uni- multivariate HR = 1.37; 95% CI: 0.83–2.25), though not significantly and with a moderate heterogeneity.

**Conclusions:**

The prognostic significance of serum p53-AAbs in ovarian cancer was diverging according to uni or multivariate models used. Since the results of this work were based on only few investigations, large prospective studies are needed to better define the role of antibody immunity against p53.

## Introduction

Epithelial ovarian cancer is the most lethal and aggressive gynaecological cancer and the fourth common cause of female cancer death in western/developed countries [1–3]. Due to confusing symptoms and no screening for early detection [4], most of ovarian cancers (~75%) are diagnosed at advanced stage (International Federation of Gynaecology and Obstetrics, FIGO, stage III-IV) of the disease [5]. Despite modern management with upfront surgery with optimal tumour debulking and subsequent adjuvant platinum based chemotherapy (CT) in combination with taxanes or neoadjuvant CT and subsequent cytoreductive surgery, the 5-year survival rate is still around 40%, [6,7]. Furthermore, about 60–70% of ovarian cancer patients after completion of primary therapy, will develop recurrence within 18 months [5, 8]. Some validated ovarian cancer prognostic factors are FIGO stage (III-IV) at diagnosis, performance status, volume of residual disease after primary surgery and histological sub-type (serous); other additional factors are older age and high-volume ascites [4,8]. Nonetheless, personalized ovarian cancer treatment is still a future challenge and no biomarkers currently exist to identify sub-groups of patients who will benefit from chemotherapy. Serologically detectable p53 autoantibodies (p53-AAbs) are a product of a spontaneous and early humoral immune response of the host against the accumulation of an antigenic mutated p53 protein in tumour cells [9]. p53-AAbs can be detected also in tissues, ascites, and other body fluids beside serum [10]. In ovarian cancer patients p53-AAbs are found generally in 20–40% of serum samples and are associated with advanced (FIGO III-IV) stages [11, 12]. Mutation or loss of *TP53* gene function due to alterations in its nucleotide sequence at the somatic level, is the most frequent genetic alteration in ovarian cancer and has been observed in 60–80% of both sporadic and familial cases [13]. The abundance in *TP53* genetic abnormalities has been associated to DNA damage increased sensitivity in the in the fallopian tube secretory epithelial cells [14]. In particular, in advanced/high-grade serous (HGS) ovarian cancers, *TP53* somatic mutations are an early hallmark, with a frequency above 95% [15, 16]. Many studies have investigated the presence of p53-AAbs in ovarian cancer for a diagnostic purpose [17], as well as in other types of cancers [18], suggesting its potential role as a screening biomarker especially in association with: 1) other early ovarian tumour markers, i.e. Carbohydrate Antigen 125 (CA-125) and Human Epididymis Protein 4 (HE4), to increase early diagnostic sensitivity; 2) imaging/radiological screening in high-risk populations [19, 20]. To date, the prognostic significance of p53-AAbs in ovarian cancer has given controversial results.

This paper focuses on the prognostic role of serum p53-AAbs in ovarian cancer after a critical and systematic review of the literature investigating the associations between clinical-pathological parameters and p53-AAbs over the last 20 years. Our goal was to elucidate the association between the clinical outcome of ovarian cancer patients and the serologically detectable immune response against p53 overexpressed by the tumour. Overall survival (OS) was the primary outcome, and disease free survival (DFS) was the secondary outcome. Moreover, we investigated the associations between p53-AAbs and baseline tumour characteristics.

## Materials and Methods

### Literature Search

PUBMED, EMBASE, Cochrane library and Web of Science databases were comprehensively searched to identify eligible studies on the association between serum p53-AAbs and ovarian cancer prognosis, including OS, DFS, relapse free survival (RFS) and progression free survival (PFS). Furthermore, reported associations between serum p53-AAbs and baseline tumour characteristics were also commented. All articles were extracted by May 29, 2015. In order to search and include all potential studies, we applied various combinations of the following medical subject headings and key words in order to hold high sensitivity: p53 autoantibodies, or serum p53 autoantibodies, or p53-AAbs, or serum autoantibodies, or p53 immunity, or anti-ovarian antibodies; ovarian cancer, or ovarian, or ovarian tumour; survival, or disease free survival, or prognosis, or outcome, or clinical. As a search limit, only studies published in English and concerning humans were included. In addition, references of other narrative and systematic reviews were checked for relevant articles.

### Eligibility criteria

All the retrieved records were independently screened by two distinct reviewers. Disagreements were resolved by consultation with a third reviewer. Firstly, all irrelevant records, reviews, case reports, studies on animals or cell lines, and studies on other cancers were excluded in addition to all papers in which presence of serum p53-AAbs was assayed only for a diagnostic purpose (early detection of ovarian cancer). Eligible studies meeting the following criteria were included to evaluate associations between p53-AAbs and ovarian cancer outcome: (1) proven diagnosis of ovarian cancer; (2) serum or plasma p53-AAbs detection by using multiple methods. Detection of autoantibodies against p53 in tissue samples by immunohistochemistry (IHC) techniques was not included, as well as evaluations of p53-AAbs in ascitic fluid or in other non-blood derived biological fluids; (3) reported associations with circulating p53-AAbs and survival data, including OS, DFS, progression free survival (PFS), and relapse free survival (RFS), in both univariate and/or multivariate analyses. Moreover, reported associations with circulating p53-AAbs and baseline tumour characteristics, i.e. p53 tumour overexpression, FIGO stage disease, tumour differentiation grade, histological subtypes, and residual tumour were also commented.

### Data extraction

The following data from collected studies were independently extracted by two observers (MG; MM): last name of first author, publication year, country, definition of ovarian cancer diagnosis, age, ethnicity, number of patients enrolled, method for p53-AAbs detection, p53 tumour expression, FIGO stage, tumour grading, residual tumour, tumour histotype, cut-off values, statistical tests data (contingency tables, Kaplan-Meier, Cox models) in univariate and/or multivariate analysis with hazard ratio (HR) or relative risk (RR), 95% confidence interval (CI) and *p* values. When the above information were not reported in the original study, the items were treated as “Not Available (NA)”. Multiple studies published by the same author(s) were checked for overlap of included case subjects. Inconsistencies in the research process, they were solved by discussion. The quality of the included studies was assessed by the Newcastle-Ottawa Scale (NOS) (http://www.ohri.ca/programs/clinical_epidemiology/oxford.asp). If a study did not clearly mention one of these key points, we considered that the point was not covered in the study, and the results may have underestimated the reported characteristics.

### Statistical analysis

To estimate the association between the presence of serum p53-AAbs (+p53-AAbs) and/or the absence of p53-AAbs (–p53-AAbs) on survival of ovarian cancer patients, only studies reporting univariate and/or multivariate HRs or RRs, corresponding 95% CI and *p* values, were considered. If both multivariate and univariate analyses were present, we chose the former. We provided estimations from given data (Kaplan-Meier curves) when these statistical variables were not available in an article, using methods reported by Tierney *et al*. [21]. Overall survival was the primary outcome, and DFS was the secondary outcome. We decided a priori to run random effect models to calculate the pooled HRs estimated and 95% CIs. Heterogeneity between studies was evaluated by means of tau-squared (τ^2^) and inconsistency index (I^2^) statistics. The heterogeneity (I^2^<25%: no heterogeneity; I^2^ = 25–50%: moderate heterogeneity; I^2^>50% or *p*>0.01: large or extreme heterogeneity) [22]. To verify the presence of publication bias, funnel plots and Egger’s linear regression test were performed [23].

The robustness of the combined results was assessed by sensitivity analysis in which studies were removed one by one each time and the pooled HR was recalculated after the exclusion to identify the studies causing considerate fluctuation of HR estimates. All the *p* values were two sided, and *p*<0.05 was considered significant, except for Egger's test where we considered a p<0.1 as statistically significant. All statistical analyses were performed using STATA 10.1 (STATA Corp., college Station, TX).

## Results

### Search results and study selection

**Fig 1:**
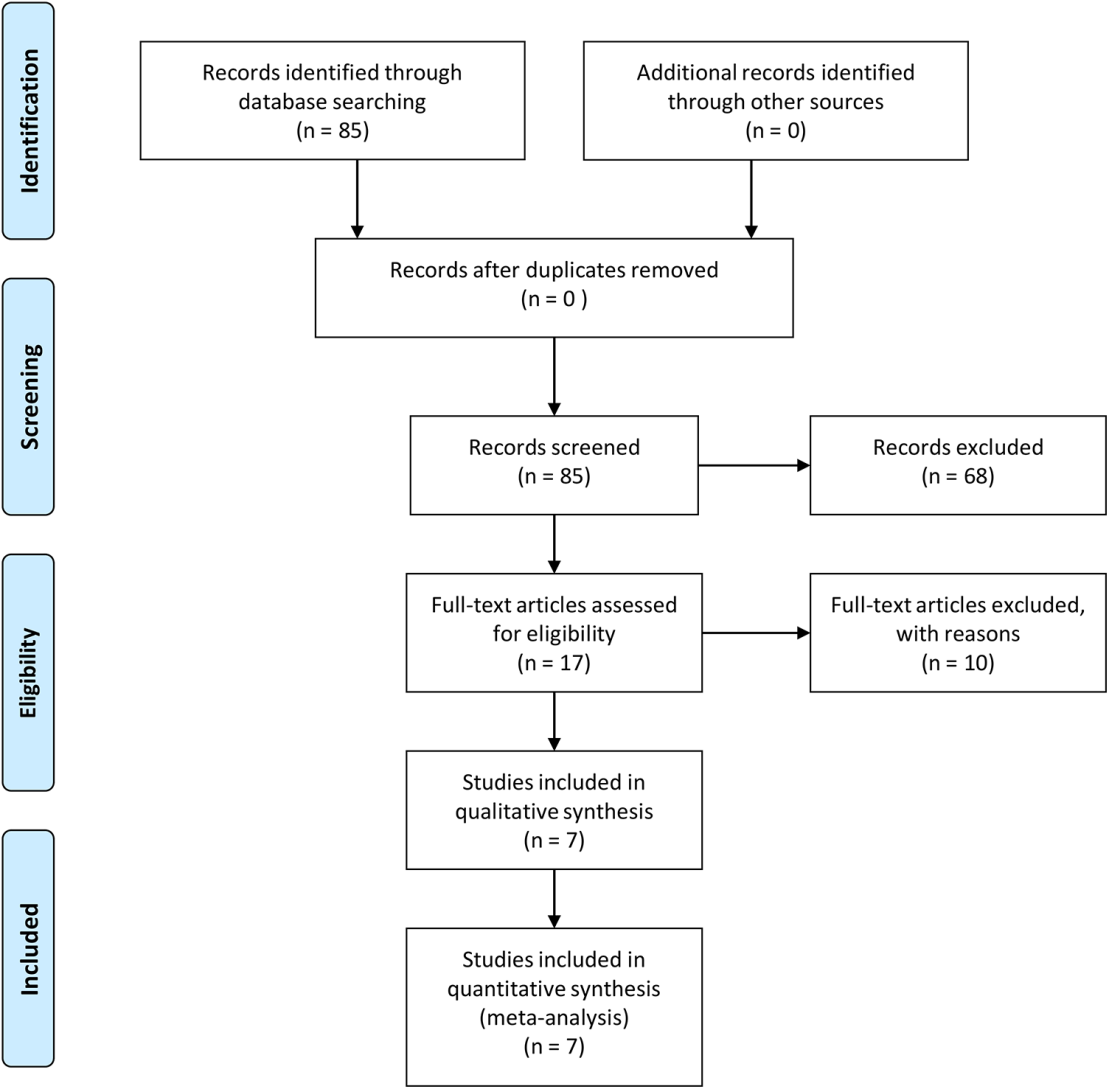
PRISMA Flow chart describing the search for relevant studies used in this meta-analysis.

By means of the above described search strategies, we initially identified 85 articles of which 68 were discarded after reading abstracts and/or full text. The remaining 17 papers were critically reviewed (systematic review). The flowchart of search strategy for articles is presented in Fig 1 and S1 Checklist. The main clinical-pathological characteristics of the 17 studies reporting associations between circulating p53-AAbs and ovarian cancer outcome are shown in Table 1.

**Table 1:** Summary of clinical-pathological characteristics of studies reporting associations between +p53-AAbs and survival in ovarian cancer.

Study, publication year	Country	N. of patients	+ p53-AAbs %	Median age, y (range)	Follow-up, m (range)	Tumour histotype	FIGO stage	Tumour Grading	Tumour Residual	p53-AAbs Method	Survival	Analysis	NOS^1^ score
Gadducci et al., 1996	Italy	30	33%	60	NA	S 20, U 4,	I 8, II 1,	G1 4,	<2 cm 6,	Dianova	DFS ↓	Fisher’sexact test	
[24]				(37–81)		M 2, E 2,	III 13, IV 8	G2 8,	>2 cm 15				
						Mx 1		G3 18					
Angelopoulou et al., 1996	Canada	174	24%	56	34	S 93, O 45,	I-II 39,	G1 51,	NA	No	DFS ↑	Multi	8
[25]				(14–88)	(1–174)	NA 36	III-IV 102,	G2 39,		commercial	OS ↑		
							NA 33	G3 54,		(TRI)			
								NA 30					
Marx et al., 1997	Germany	130^2^	41%	60	NA	S 100, E 23,	I 12, II 20,	G1 3,	R0 16,	Oncogene	DFS ↓	Fisher’sexact test	
[26]				(27–87)		M 2, U 2, C 3	III 81,	G2 64,	<2 cm 25,			or Chi- square	
							IV 17	G3 53,	≥ 2 cm 12				
								G4 10					
Gadducci et al., 1998	Italy	40	37%	62.5	NA	S 24, U 9,	III 25,	G1 5	NA	Immunotech	DFS ↓	Fisher’sexact test	
[27]				(36–85)		M 7	IV 15	G2 14,				or Chi- square	
								G3 21					
Mayerhofer et al., 1999	Austria	33	36%	<50 y 11,	NA	S 15, M 4,	Ia 4, Ic 4,	G1 12,	NA	RD	OS ↓	Uni	
[28]				>50 y 22	(8–52)	U 4, C 3, O 7	II 6, III 19	G2 5,					
								G3 16					
Gadducci et al., 1999	Italy	86	21%	61	48	S 52, U 12, M	I-II 30,	G1 23,	<2 cm13,	Pharmacell	PFS ↓	Uni	9
[29]				(23–92)	(10–71)	8, E 7, O 7	III-IV 56	G2 24,	>2 cm 43^3^		OS ↓		
								G3 39					
Vogl et al., 1999	Germany	83	46%	59	24	S 62, M 7,	I 9, II 10,	G1 9,	NA	Dianova	RFS ↓	Multi	9
[12]				(23–89)	(1–44)	E 6, O 8	III 52,	G2 27,			OS ↓		
							IV 11	G3 46					
Vogl et al., 2000	Germany	113	19%	61	22	S 68, O 45	I 23, II 13,	G1 13,	Yes, NA	No	RFS ↓	Multi(OS)	8
[30]				(21–89)	(1–68)		III 62,	G2 41,		commercial	OS ↓		
							IV 15	G3 57				NA	
Abendstein et al., 2000	Austria	113	25%	64	36.4	S 60, M 32,	Ic 13, IIc 8,	G1 9,	R0 44,	Dianova	DFS	Uni	
[31]				(24–79)	(NA)	E 9, C 3, U 9	III 78,	G2 57,	<2 cm 27,		OS	NA	
							IV 14	G3 44	>2 cm 42				
Numa et al., 2001	Japan	30	27%	62 +p53-AAbs,	NA	S 19, M 6,	I-II 9,	NA	NA	No	OS ↓	Uni	6
[32]				53 –p53-AAbs		O 5	III-IV 21			commercial			
Marx et al., 2001	Germany	99^4^	26%	59	NA	S 78, E 15,	I 9, II 12,	G1 2,	R0 13,	Dianova	DFS ↓	Fisher’sexact test	
[33]				(27–87)		M 1, U 2, C 3	III 66,	G2 50,	<2 cm 21,			or Chi- square	
							IV 12	G3 38,	≥2 cm 12				
								G4 9					
Hogdall et al., 2002	Denmark	193	12%	60	NA^5^	S 110, P 13,	I 50, II 3,	NA	Rx 121	No	OS	Multi	
[34]				(35–79)		O 70	III 111,			commercial		NA	
							IV 19						
Goodell et al., 2006	USA	104	24%	59	22	S 79, M 7,	I 20, II 4,	NA	NA	No	OS ↑	Multi	9
[35]				(34–89)	(0–75)	E 4, C1,	III 67,			commercial			
						U 5, O 8	IV 13						
Leffers et al., 2008	Holland	233	16%	<59 123,	37.4	S 125, O 108	I-II 76,	G1-G2	<2 cm	Dianova	OS	Uni	
[36]				≥59 110	(27.5–47.3)		III-IV 156	103, G3-	132,				
								G4 114	≥2 cm 89				
Tsai-Turton et al., 2009	USA	130	25%	NA	NA	NA	I 10, II 7,	G1-G2	NA	Calbiochem	OS	Uni	
[11]							III 79, IV	14, G3-					
							34, 14 NA	G4 116					
Anderson et al., 2010	USA	90	32%	NA (42–71)	36.8	S 60, E 10, C	NA III-IV	G3 60^6^	≤1 cm36,	No	OS ↑	Multi	9
[37]					(25^th^ perc.,	10, M 10	(S 57)		>1 cm 6,	commercial			
					22.2; 75^th^				NA 18^5^				
					perc., 68.6)								
Hafner et al., 2013	Germany	10	80%	NA	NA	NA	NA	NA	<1cm	Steinbeis	PFS ↓	NA	
[38]													

↓: diminished

↑: increased; p53-AAbs: p53 autoantibodies; y: years; m: months; FIGO: International Federation of Gynecology and Obstetrics; NOS: Newcastle-Ottawa Quality Assessment Scale; perc.: percentile; Uni: univariate; Multi: multivariate; NA: Not Available; TRI: Time-resolved immunofluorimetric assay; S: Serous; U: Undifferentiated; M: Mucinous; C: Clear cell; E: Endometroid; Mx: Mixed; P: Papillary; O: Other; G1: well differentiated; G2: moderately differentiated; G3: poorly differentiated; G4: undifferentiated;Rx: Residual tumour size indefinite

^1^NOS score (range, 1–9) was assigned only to the included studies

^2^53/130 patients enrolled prior to first surgery

^3^all these patients (N = 43) had III-IV FIGO stage

^4^46/99 patients enrolled prior to first surgery

^5^Follow-up data available for patients dead and alive

^6^data available only for patients with serous tumour histotype.

Six studies were not considered due to lack of univariate and/or multivariate data [31, 38], and statistical evaluations through contingency tables [24, 26, 27, 33]. Associations between survival and p53-AAbs in univariate and/or multivariate analyses were reported in the remaining 11 studies [11, 12, 25, 28–30, 32, 34–37]. Among them, 4 were excluded for insufficient data on survival [11, 28, 34, 36]. Finally, 10 studies were not included and 7 [12, 25, 29, 30, 32, 35, 37] were considered eligible for the meta-analysis, 7 for OS [12, 25, 29, 30, 32, 35, 37] and 4 for DFS [12, 25, 29, 30]. These studies were conducted in 5 countries: Canada, Italy, Germany, Japan and USA (Washington State [35] and Massachusetts [37]) and published between 1996 and 2010. The total number of patients included were 583 (356 for DFS analysis), with sample sizes ranging from 30 to 113 patients. Age ranged from 14 to 92 years. The median follow-up period ranged from 22 to 48 months. HRs and corresponding 95% CIs were derived directly from 4 articles [12, 25, 35, 37] and calculated or extrapolated from Kaplan-Meier curves in the remaining studies [29, 30, 32]. Overall included studies had a moderate or high quality NOS score. Quality assessment is shown in Table 1.

### Study characteristics

Overall serum samples were collected before surgical treatment or within 6 months from diagnosis [25]. In some studies, samples were collected also at different times during follow-up [26, 27, 33, 38]. The presence of autoantibodies against p53 was measured in plasma samples in only one study [11], while it was also evaluated in ascitic fluid in another one [31]. Autoantibodies were detected by standard sandwich ELISA method in 16 studies, by means of a commercial kit (N = 11) or a non commercial ELISA (N = 5) (Table 1). Only one article [25], reported a different method for p53-AAbs detection, the time-resolved immunofluorimetric technique (TRI) that was previously developed as an alternative”sandwich” immunoassay [39]. No proteomic techniques were reported to detect p53-AAbs in these articles. We observed frequent correlations between the +p53-AAbs and p53 overexpression, advanced stage disease and G2-G3 tumours. Evaluation of p53 overexpression by IHC in tumour tissue samples was performed in 9/17 studies, with a significant association with +p53-AAbs in 6/9 papers [11, 12, 25, 30, 32, 36] (Table 1). An association with FIGO advanced stages (III-IV *vs* I-II) and +p53-AAbs was found in 10 studies, with statistically significant associations in 6/10 [11, 12, 30, 35–37]. Moderate/poor differentiated (G2-G3) ovarian cancer tumours were found to be associated to +p53-AAbs in 6 studies, 3/6 with a statistically significant association [25, 30, 36]. Residual tumour (R≥2 cm) after primary surgery was associated to serum +p53-AAbs only in one article [29]. The most aggressive histological subtype for ovarian cancer, the serous histotype, was significantly associated to serum +p53-AAbs in two studies [11, 37]. Age above ≥50 years [12, 25], post-menopausal status [12], cytogenetic alterations [32], higher (IL-4, IL-12) and lower (IL-18) levels of interleukins [11], and breast cancer history [37], were occasionally found to be significantly associated with +p53-AAbs. Furthermore, CA-125 levels were compared to +p53-AAbs levels only in two studies [11, 34]: patients with detectable +p53-AAbs in serum had also significantly higher levels of CA-125 with respect to –p53-AAbs.

### Serum p53-AAbs and prognostic value in ovarian cancer

**Fig 2:**
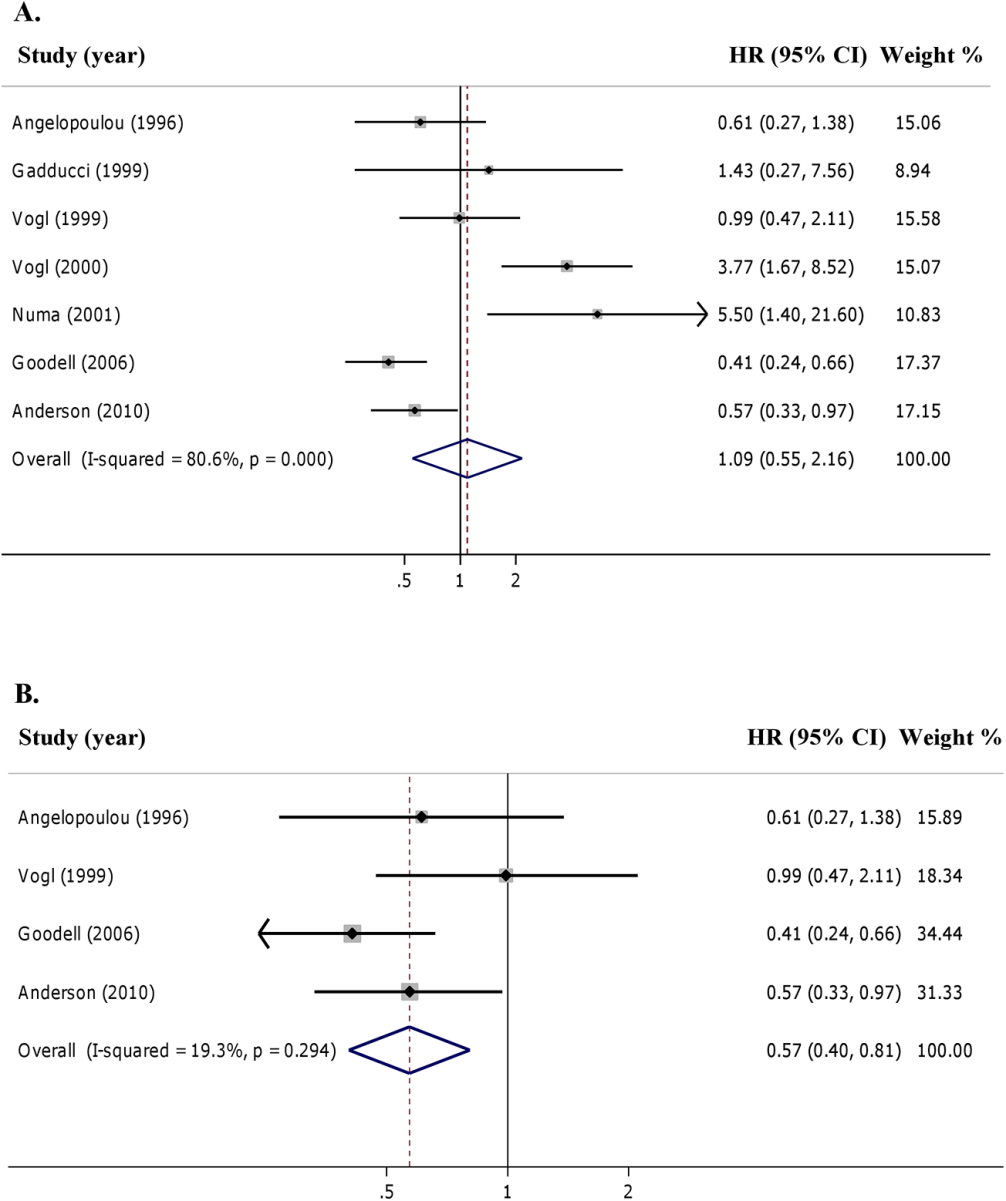
Forest plots of adjusted Hazard Ratios (HRs) for Overall Survival in ovarian cancer according to presence of p53-AAbs. **Random effect models were computed.** (**A**) Pooled analysis considering uni- multivariate HRs (7 studies); (**B**) pooled analysis with only multivariate HRs (4 studies).

Seven studies [12, 25, 29, 30, 32, 35, 37] were pooled into the final meta-analysis to define the association between serum p53-AAbs and OS. Both univariate [29, 30, 32] and multivariate [12, 25, 35, 37] estimates were included in the analysis. When the 7 eligible studies were grouped, the presence of p53-AAbs was not associated to OS (pooled HR = 1.09 for +p53-AAbs *vs* –p53-AAbs, 95% CI: 0.55–2.16) (Fig 2A), and a large heterogeneity was observed (I^2^ = 80.6%, τ^2^ = 0.629, *p*<0.001, Fig 2A). Since adjusting factors such as age, FIGO stage, histotype, and tumour grading are important variables that may influence patient’s outcome in ovarian cancer, we carried out a further analysis with studies in which an adjustment for known prognostic factors was performed. When meta-analysis was performed considering the 4 studies presenting results by multivariate estimations [12, 25, 35, 37], the +p53-AAbs was significantly associated with a better OS (pooled HR = 0.57 for +p53-AAbs *vs* –p53-AAbs, 95% CI: 0.40–0.81) (Fig 2B). No significant heterogeneity was observed between the 4 included studies (I^2^ = 19.3%, τ^2^ = 0.025, *p* = 0.294, Fig 2B).

**Fig 3:**
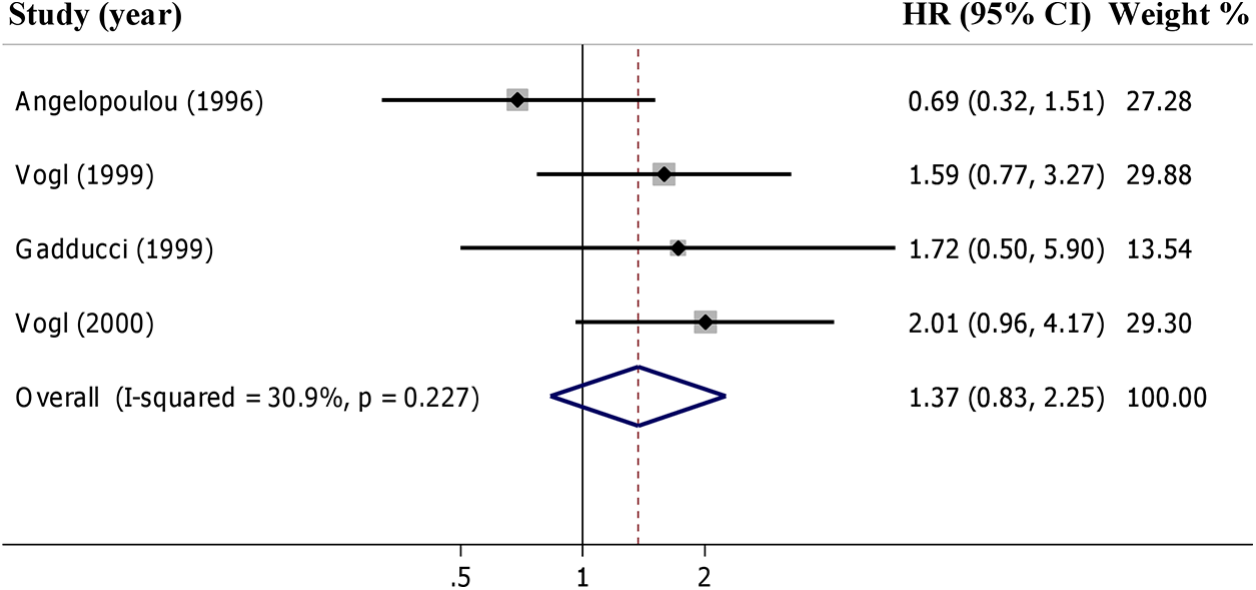
Forest plot of adjusted Hazard Ratios (HRs) for presence of p53-AAbs and Disease Free Survival in ovarian cancer according to p53-AAbs status.

Concerning DFS, the presence of p53-AAbs was associated to a worse but not statistically significant DFS when the 4 studies [12, 25, 29, 30] were pooled in the meta-analysis (pooled HR = 1.37 for +p53-AAbs *vs* –p53-AAbs, 95% CI: 0.83–2.25) (Fig 3). This meta-analysis demonstrated a moderate heterogeneity (I^2^ = 30.9%, τ^2^ = 0.080, *p* = 0.227, Fig 3). Only two studies [12, 25] presented multivariate estimates for DFS; we did not find any association with DFS in the pooled analysis (pooled HR = 1.06 for +p53-AAbs *vs* –p53-AAbs, 95% CI = 0.47 to 2.40) (not shown). It is worth noting that HR estimates were not consistent between the two studies (HR = 1.59 and HR = 0.69) with a wide heterogeneity (I^2^ = 58%, τ^2^ = 0.029, *p* = 0.276). We noticed that studies linked to a statistically significant favourable prognosis in OS [35, 37], restricted the multivariate analysis to a sub-set of ovarian cancer patients with advanced stage, serous histotype, and poor differentiated tumours (Table 2): this way, the positivity for p53-AAs increased from 24% to 30% [35] and from 32% to 42% [37].

**Table 2:** Summary of clinical-pathological parameters of patients in the studies presenting multivariate HRs included in the meta-analysis.

Study, publication year	N. of patients	N. of patients in Multivariate Analyses (%)	% FIGO Stage III-IV	% G3 Grading	% Serous Histotype	% +p53-AAbs	% of patients receiving CT	Adjustment with the following variables
Anderson et al., 2010	90	60 in OS (67%)	95%	100%	100%	42%	98%	Age, y diagnosis, FIGO stage,
[37]								laboratory batch number, PLT-based
								CT, number of CT cycles
Goodell et al., 2006	104	80 in OS (77%)	100%	NA	84%	30%	NA	Age, Stage, HER-2/neu and
[35]								topoisomerase IIα AAbs,
								CA-125 level at diagnosis
Vogl et al., 1999	83	83 in RFS & OS (100%)	76%	56%	75%	46%	77%	Age, FIGO stage, Grading
[12]								
Angelopoulou et al., 1996	174	74 in DFS (42%)	72%*	38%*	53%*	67%*	71%*	Age, FIGO stage, Grading,
[25]		98 in OS (56%)						Histotype, CT

p53-AAbs: p53 autoantibodies; FIGO: International Federation of Gynaecology and Obstetrics; PLT: Platinum; CT: Chemotherapy; NA: Not Available; G3: poorly differentiated

*% is not related to the total of patients [25].

Among the selected studies, one [25] showed a multivariate analysis limited to a sub-group of patients for OS (56%) as well as for DFS (42%), presumably for missing data (Table 2).

### Publication Bias Assessment and Sensitivity Analysis

**Fig 4:**
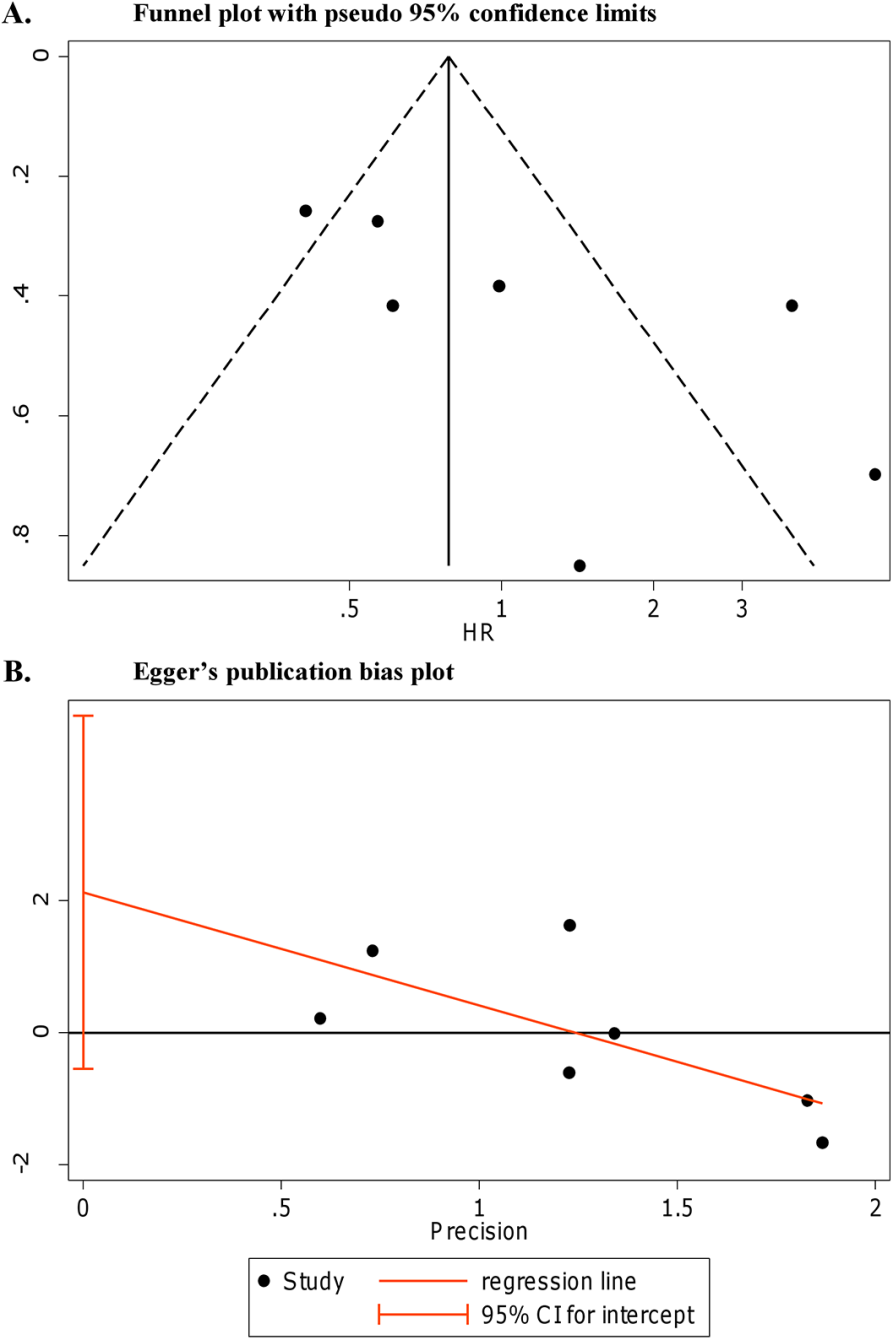
Publication bias assessment of the studies assessing p53-AAbs presence and OS in ovarian cancer. (**A**) Funnel plot for publication bias assessment of pooled OS; (**B**) Egger’s linear regression test.

Visual assessment of the funnel plot suggested some publication bias with a positive asymmetry for all the 7 studies included in pooled OS (Fig 4A).

**Fig 5:**
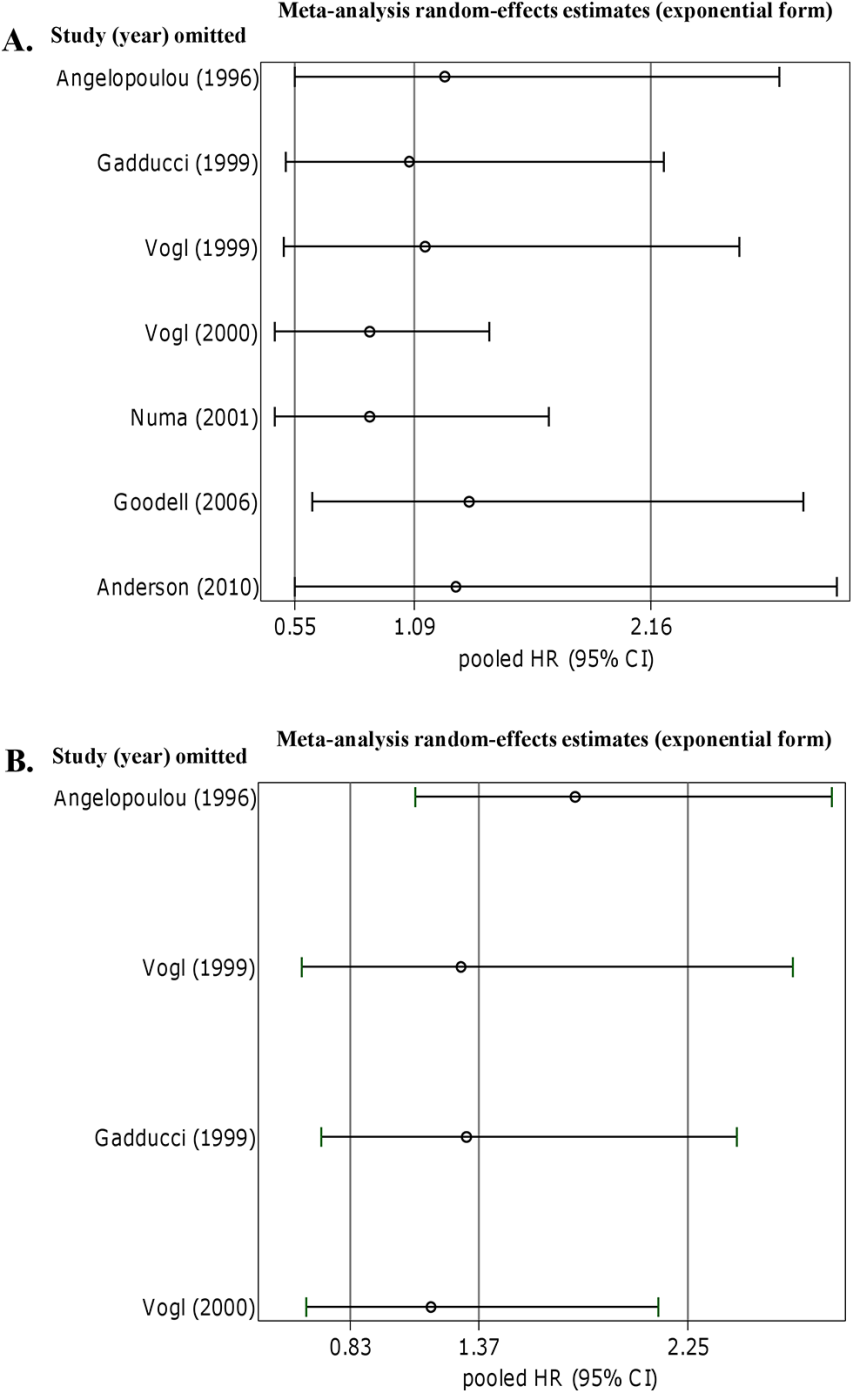
Sensitivity analysis. **The analysis was conducted by estimating the average HR in the absence of each study.** (**A**) Sensitivity analysis of all studies assessing associations between presence of p53-AAbs in ovarian cancer for OS, and DFS (**B**).

The Egger’s test showed some evidence of small study effect for pooled OS (*p* = 0.096) (Fig 4B). Sensitivity analysis was performed to evaluate the stability of results. No study was found to significantly affect either the pooled HRs for OS (Fig 5A) or the pooled HR for DFS (Fig 5B). When studies concerning p53-AAbs and OS were grouped by ethnicity excluding the only Asian study [32] by sensitivity test, the heterogeneity was still high (I^2^ = 78.1%, τ^2^ = 0.629, *p*<0.001) and the +p53-AAbs was associated with a better OS though not reaching statistical significance (pooled HR = 0.89 for +p53-AAbs *vs* –p53-AAbs, 95% CI: 0.47–1.70). Due to the low number of studies, the Egger’s test was not applied for publication bias assessment in pooled DFS. Nonetheless, the funnel plot showed some positive asymmetry (not shown). The sensitivity analysis for DFS, showed that results did not change significantly (Fig 5B).

## Discussion

This paper summarised for the first time the prognostic role of serum p53 autoantibodies in ovarian cancer. To our knowledge, this is the first systematic review and meta-analysis on this topic. The usefulness of p53-AAbs as prognostic biomarkers in clinical outcome of ovarian cancer, in particular, has been poorly investigated; the role of p53-AAbs is still controversial but critical in understanding their function in the immune surveillance of cancer [40].

The presence of circulating autoantibodies against specific tumour-associated antigens (TAA) is generally found in less than 1/3 of cancer patients, resulting in poor diagnostic sensitivity [41]. Autoantibodies reflect both changes in the primary tumour as well as effective cancer immune surveillance, and may represent candidates for immunotheraphy development if they are associated to improved prognosis [40]. Nonetheless, AAbs can be detected up to 5 years before symptomatic disease, demonstrating that the human immune system recognizes the autologous TAAs as “non-self” producing an earlier humoral response in the patients [42]. Combination of panels of TAAs to detect multiple different and specific AAbs is the current goal to increase their diagnostic potential [20]. Anyway, recent evidences suggest that serum p53-AAbs can be considered as biomarkers to detect many types of cancer, including ovarian cancer [17]. p53-AAbs are usually IgG indicating a secondary response after a prolonged immunization process probably before the diagnosis of the malignancy [43]. The evidence that in healthy subjects p53-AAbs are extremely rare [17] explains the concept that cancer cells carrying mutations in *TP53* gene and with p53 over-expression are the source of a self-immunization process. However, considering that only a subset of cancer patients (~20–50%) carrying *TP53* somatic mutations have detectable p53-AAbs, genetic variants in *TP53* alone are probably not sufficient to trigger the p53-AAbs secretion, but mutations of p53 regulators and non-mutative pathways are probably engaged as well [9]. Furthermore, in ovarian cancer the correlation between p53 accumulation, tumour grade and p53-AAbs detection has not always been found [44]. Overall, these observations suggest that the biological and immunogenetic background of individuals, such as the set of major histocompatibility complex (MHC) classes I and II molecules, should be considered in the induction of an anti-p53 specific humoral response [36, 44–47].

As represented in Fig 1, to examine the correlation between p53-AAbs and survival, only 7 studies were eligible for meta-analysis from 17 candidate articles (Table 1), via systematic review. These results confirmed the suggestion that the presence of autoantibodies against p53 in the serum of ovarian cancer patients has been poorly investigated in the last 20 years.

To define the prognostic role of p53-AAbs as biomarkers for clinical utility, they must be independent of known clinic-pathologic criteria, and well-established validation assays tested in large cohorts of patients and control populations are also required. Moreover, an accurate clinical history of patients and long term follow-up are necessary. Among the 17 studies (Table 1) initially selected, 53% (9/17) of them did not carry out an investigation in a control group of subjects, i.e age matched healthy donors and/or patients with cystadenomas and/or benign gynaecological diseases [12, 25, 27, 29, 31, 33, 35, 36, 38]. This point could affect the quality of p53-AAbs detection when a non commercial assay was used. Moreover, blood samples should be collected prior to treatment (first surgery and/or chemotherapy) even if an immunological memory exists. Finally, data should be analyzed also considering sub-groups of patients who received uniform therapies. We observed that in different studies the presence of p53-AAbs was significantly associated to overexpression of p53 in the tumour (67%), to III-IV stages (60%), and to G2-G3 tumours (50%). Among the 7 articles [12, 25, 29, 30, 32, 35, 37] selected for meta-analysis, in 5 [25, 30, 32, 35, 37] a non commercial ELISA test was used to measure serum p53-AAbs and a matched control group was considered only in 3 studies [30, 32, 37]. Data on the chemotherapy treatment were not reported in 2 papers [35, 32] and not described in detail in another one [25].

Ten studies showed a tendency toward a poor outcome (Table 1): 8 articles were associated to shorter DFS/PFS/RFS [12, 24, 26, 27, 29, 30, 33, 38] and 5 to decreased OS [12, 28–30, 32]. Three studies showed an association with a favourable outcome [25, 35, 37]. The presence of serum p53-AAbs was statistically significantly associated to a reduced DFS in ovarian cancer only in univariate analysis in 2 studies [12, 25]. In multivariate analysis, +p53-AAbs failed to be an independent prognostic factor in both studies, one was associated to a favourable DFS [25]. However, HR estimates were not consistent between the univariate and multivariate analyses in this paper [25] in both OS and DFS. In the final pooled analysis with 4 eligible studies [12, 25, 29, 30], we found an association with a reduced DFS and a moderate heterogeneity.

Concerning OS, no associations between p53-AAbs and survival were found in 4 studies [11, 31, 34, 36]; anyway, data about OS in univariate analysis (log-rank *p* value) were reported for 2 articles [11, 36]. As described in the Results, for the final meta-analysis, 7 studies [12, 25, 29, 30, 32, 35, 37] were eligible for OS evaluation. Only 5 studies [12, 25, 30, 35, 37] presented results about multivariate estimations. Detectable serum p53-AAbs were significantly associated to survival after adjustment for ovarian cancer main prognostic factors in 3 studies [30, 35, 37], even if, one [30] did not report the estimated HR and 95% CI. Three studies [25, 35, 37] were associated to a better outcome, however, one of these did not reach significance [25]. When we considered the 7 studies overall, we found a large heterogeneity and no association with the presence of the p53-AAbs and OS. As suggested by the Egger’s test and the funnel plot, we found evidences for publication bias and for some small study effect [32], which could explain the large heterogeneity in the OS analyses. Intriguingly, when the 4 studies reporting the multivariate HRs estimations [12, 25, 35, 37], adjusted for known prognostic factors that may have influenced survival were pooled together, no significant heterogeneity was detected, and the presence of autoantibodies was significantly associated to a better OS. In particular, we observed that the latest studies restricted the survival analyses only to patients with advanced stage [35] and serous histotype [37] and adjusted the multivariate analyses for multiple factors (Table 2), finding an association with a better survival. The stratification used seemed logical since the presence of p53-AAbs proved to be associated to these pathological parameters [11, 12]. Furthermore, authors adjusted multivariate analyses with a more complete panel of variables, compared to the other 2 studies. Some biologically plausible mechanisms may explain the p53-AAbs potentially direct or indirect protective role in ovarian cancer. p53-AAbs appearance in serum is a product of a natural immunization process detectable only in a subset of patients, particularly with advanced stage disease. An anti-p53-specific IgG autoantibodies may induce amplification of specific p53-T-cell memory response [48], but the function of these autoantibodies is still unknown [41].

## Conclusion

This review included all studies reporting univariate or multivariate estimates of ovarian cancer prognosis linked to p53-AAbs. Our study suggested that serum p53-AAbs have a controversial prognostic role in ovarian cancer, although their presence was significantly associated to an improved OS only at multivariate analyses. Autoantibodies were associated with a worse, although not significant DFS, while no association was observed including only multivariate HRs. Nonetheless, the following limitations should be considered to interpret meta-analyses results: (1)- the low number of studies included in the meta-analysis, especially for DFS; (2)- univariate and multivariate HRs were pooled together and this may explain the wide heterogeneity observed; (3)- presence of autoantibodies against p53 was determined by means of different types of ELISA assays (commercial or not) thus, misclassification is possible; (4)- the p53-AAbs detection was not validated in a matched control group in most of eligible studies; (5)- Caucasian/white female patients who may limit the comparison of our results to other populations.

However, the meta-analysis has several strengths, including the homogeneity of ovarian cancer diagnosis, the prospectively collected OS and DFS data, and the univariate and multivariate estimations of HRs. Furthermore, measures of p53-AAbs levels were based on blood samples collected shortly after diagnosis and prior to primary surgery. This review shows that, to date, p53-AAbs have had a limited clinical application. Our conclusions are based on few investigations thus, should be considered carefully. Further researches in large patient’s cohorts are needed to explore the role of the natural immunity process against the oncogenic p53 protein in ovarian cancer.

## Supporting Information

S1 Checklist PRISMA Checklist. (DOC)
